# A Real-Time Dynamic Warning Method for MODS in Trauma Sepsis Patients Based on a Pre-Trained Transfer Learning Algorithm

**DOI:** 10.3390/diagnostics16020270

**Published:** 2026-01-14

**Authors:** Jiahe Wen, Guanjun Liu, Panpan Chang, Pan Hu, Bin Liu, Chunliang Jiang, Xiaoyun Xu, Jun Ma, Guang Zhang

**Affiliations:** 1 Academy of Military Sciences of the People’s Liberation Army Institute of Systems Engineering, Tianjin 300161, China; 19146412338@163.com (J.W.); guan_jun0502@163.com (G.L.); chenzxxy@163.com (X.X.); 2 Trauma Medicine Center, Peking University People’s Hospital, Beijing 100044, China; drchang@yeah.net (P.C.); panhu_1989@163.com (P.H.); 3 Department of Infectious Diseases, Characteristic Medical Center of Chinese People’s Armed Police Forces, Tianjin 300162, China; iamicehe@163.com; 4 School of Control Science and Engineering, Tiangong University, Tianjin 300387, China; chunliang95@foxmail.com

**Keywords:** multiple organ dysfunction syndrome, sepsis, trauma, pre-train, transfer learning, interpretable model

## Abstract

**Objectives:** Multiple organ dysfunction syndrome (MODS) is a serious, prognostically poor complication in trauma sepsis. We developed an interpretable, multicenter-validated prediction model to enable early, individualized risk assessment and guide timely care. **Methods:** Using MIMIC-IV and eICU data, we built a pre-trained transfer-learning model with a separation processing strategy and assessed interpretability with SHAP. **Results:** Internal validation included 700 MIMIC-IV patients; external validation included 110 eICU patients. Across 6-, 12-, and 24-h prediction windows, the best pre-trained model achieved an average AUC of 0.906. Notably, fine-tuning on only 100 trauma sepsis cases (3.6% of the training set) still yielded an AUC of 0.846, surpassing the non-pre-trained model by 0.165. SHAP analysis further revealed that platelet count was one of the most important variables contributing to MODS prediction. **Conclusions:** Overall, the pre-trained MODS model demonstrated robust discrimination, generalizability, and clear interpretability in both internal and external validations, highlighting its portability and clinical potential for early identification of high-risk trauma sepsis patients.

## 1. Introduction

Sepsis is a systemic inflammatory response syndrome caused by infection, characterized by a dysregulated host immune response, which leads to tissue damage and organ dysfunction, and in severe cases, death [1]. In 2017, an estimated 48.9 million incident cases of sepsis occurred worldwide, with approximately 11.0 million sepsis-related deaths, representing about 19.7% of all global deaths [2]. Traumatic injury is also a major global health burden, causing about 4.4 million deaths annually (nearly 8% of all deaths) and substantial disability [3]. In trauma patients, disruption of the skin and mucosal barriers, extensive tissue damage, and hypoperfusion can facilitate pathogen invasion and compromise host defenses, predisposing patients to infection and sepsis [4,5]; notably, sepsis has been reported to contribute to 10% of trauma-related deaths [6].

When sepsis-related infection enters the bloodstream, it triggers a systemic immune response, leading to the release of large amounts of inflammatory mediators. This cascade causes vasodilation, hypotension, and inadequate organ perfusion, eventually progressing to MODS. MODS refers to the dysfunction of two or more organ systems resulting from a systemic inflammatory response induced by severe infection, trauma, or burns. It represents a critical stage in the progression of sepsis and is one of its leading causes of mortality [7]. Since the release of the Sepsis-3 in 2016, which characterized sepsis as life-threatening organ dysfunction resulting from a dysregulated host response to infection, research has increasingly focused on MODS as a central complication [8]. The pathogenesis of MODS is complex and typically involves multiple interacting pathological factors. Without timely intervention, it may progress to irreversible multiple organ failure (MOF), which carries an extremely high mortality rate and currently lacks effective targeted therapies [9,10].

Timely prediction of MODS development in trauma sepsis patients can assist clinicians in early identification of high-risk individuals and implementation of targeted interventions, thereby delaying or mitigating disease progression, improving patient outcomes, and optimizing medical resource allocation. Currently, various organ dysfunction scoring systems are employed to assess and identify MODS in ICU patients. Among them, the Sequential Organ Failure Assessment (SOFA) score is the most widely used due to its robust clinical validation and broad acceptance. However, existing scoring systems still have limitations. They are primarily designed to assess the current clinical status and lack dedicated mechanisms for predicting the development of MODS. Additionally, these systems rely solely on physiological parameters measured at a single time point, without incorporating temporal dynamics that reflect trends and fluctuations in the patient’s condition.

At present, many studies have focused on the early warning and mortality prediction of MODS in ICU patients. For instance, Liu Chang et al. (2023) developed a model using deep neural networks and XGBoost based on the MIMIC-III and MIMIC-IV databases to predict MODS risk 12 h in advance [10]. Zhang Guang’s team (2022) employed LGBM and Adaboost for real-time dynamic prediction of MODS in ICU patients [11]. In the same year, Xiaoli Liu et al. proposed a mortality prediction model for elderly ICU patients with MODS, demonstrating superior performance compared to traditional scoring systems [12]. However, none of these studies specifically targeted trauma sepsis patients, a subgroup with distinct etiological, clinical, and prognostic characteristics [13].

Pre-training has achieved substantial success in natural language processing (NLP) by leveraging large-scale data to learn transferable representations, thereby reducing dependence on annotated data and improving generalization to new tasks [14,15]. Recently, analogous strategies have been extended to the medical domain. In 2020, Yikuan Li et al. pre-trained a model on diagnostic code histories to predict 301 clinical conditions, improving performance by 8.0–13.2% over baseline methods [16]. In 2023, Huang et al. applied a similar approach to predict outcomes of non-accidental trauma [17]. Despite these advances, existing studies largely mirror NLP-based frameworks and do not fully exploit structured clinical test data, indicating a gap in deeply integrating such data into predictive modeling.

This study proposes a real-time MODS prediction model based on a pre-training strategy, capable of simultaneously predicting the risk of MODS in trauma sepsis patients within 6, 12, and 24 h. The model underwent internal validation using the MIMIC-IV database and multicenter external validation using the eICU database. Ablation experiments were conducted to assess the contribution of key components, including the separation of high- and low-frequency data and the pre-training mechanism. Additionally, SHAP-based interpretability analysis was performed to quantify the impact of each input feature on the prediction outcomes, thereby enhancing the model’s clinical transparency and reliability.

## 2. Materials and Methods

### 2.1. Study Population

We analyzed clinical data from two large-scale public archives. The primary dataset, MIMIC-IV v2.2 [18], spans 2008–2019 and includes detailed temporal and therapeutic information for more than 190,000 patients. The eICU v2.0 database [19], which covers data from 2014–2015 and comprises a multi-institutional sample of 200,856 U.S. patients, was subsequently used to externally validate the model’s performance.

**Figure 1:**
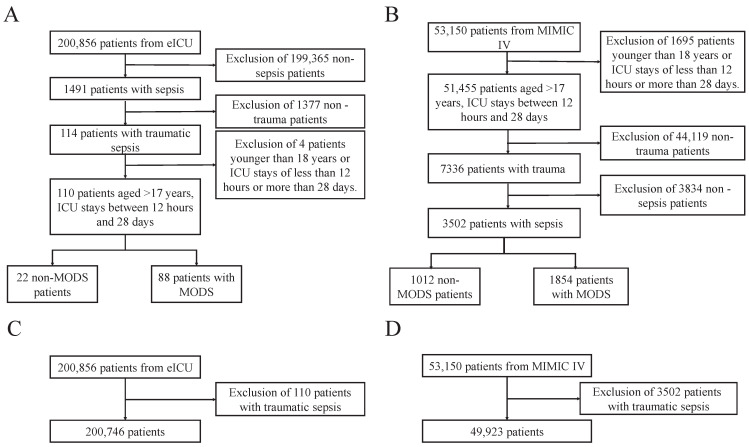
Flowchart for patient inclusion. (**A**,**B**) Data screening process for trauma sepsis patients utilized in the MODS model, where (**A**) are eICU and (**B**) are MIMIC-IV; (**C**,**D**) The patient data screening process for pre-trained models, where (**C**) are eICU and (**D**) are MIMIC-IV.

In alignment with prior research [11,20], we included adult patients with traumatic sepsis and ICU stays of 12 h to 28 d to ensure adequate training data while limiting the influence of prolonged ICU stays on MODS analyses. Under Sepsis-3.0 criteria [21], 3502 eligible cases were identified in MIMIC-IV and 110 in eICU. For pre-training, all traumatic sepsis patients were excluded; the remaining ICU population comprised 49,648 admissions in MIMIC-IV and 200,746 in eICU (Figure 1).

### 2.2. Definitions

Sepsis: According to the Sepsis-3 definition, sepsis is diagnosed when there is a clear or suspected infection accompanied by an acute increase of 2 or more points in the SOFA score [21].

MODS: Patients were classified as having MODS if they had SOFA scores of 2 or higher in at least two organ systems [12,13,22].

Observation window: To comprehensively capture the temporal characteristics of physiological parameters while minimizing the exclusion of patients due to an excessively long observation window, the model’s observation window was set to 4 h based on previous research [14,21,23].

Prediction window: The model adopts a multi-label classification strategy, enabling simultaneous prediction of whether a patient will develop MODS within the next 6, 12, or 24 h.

### 2.3. Overall Flow Chart for MODS Prediction

**Figure 2:**
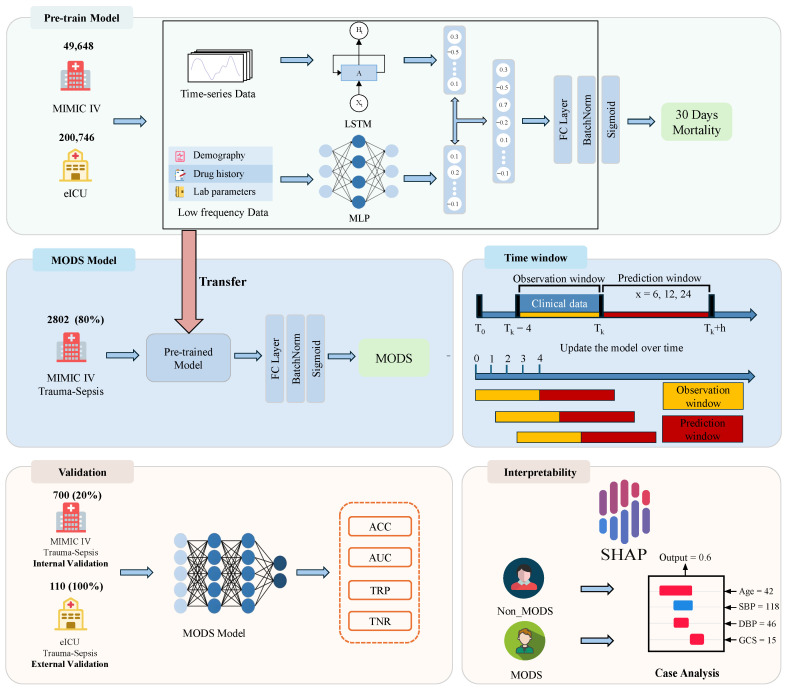
The overall flow chart of MODS prediction.

Figure 2 shows the process of prediction of the MODS probability.

1. Pre-train Model: Data of all ICU patients except trauma sepsis patients were collected from the MIMIC-IV and eICU databases, totaling 250,394 cases for model pre-training. The original data are divided into high-frequency time-series data and low-frequency data. These are input into the LSTM and the multilayer perceptron (MLP) models, respectively, to predict the risk of death within the next 30 days (“Pre-train Model” section of Figure 2);
2. MODS Model: Based on the pre-trained model, 80% of the MIMIC-IV trauma sepsis patient data were used for fine-tuning, resulting in the final real-time MODS prediction model (“MODS Model” section of Figure 2). This model employs a 4-h observation window and predicts the occurrence of MODS within the next 6, 12, and 24 h (“Time window” section of Figure 2);
3. Validation: The remaining 20% of the MIMIC-IV data and all eICU trauma sepsis patient data were used for internal and multicenter external validation of the model (“Validation” section of Figure 2);
4. Interpretability: The SHAP method is used to perform interpretability analysis on the model to reveal the impact of key features (“Interpretability” section of Figure 2).

### 2.4. Data Pre-Processing

To optimize dataset reliability, we first removed invalid records and outliers guided by clinical expertise. For data integrity, missing entries were imputed using either mean or extreme values [24]. To mitigate the impact of varying feature scales, we normalized continuous data via Z-score standardization [25], calculated as:

(1) $$z=\frac{x-\mu }{\alpha }$$

where *x* is the original value, μ is the mean, and α is the standard deviation.

Finally, categorical variables were mapped to numerical forms using One-Hot encoding to facilitate algorithmic processing [26].

### 2.5. Feature Selection

The model includes 41 input features in total, encompassing vital signs (11 features), laboratory biochemical markers (24 features), demographic characteristics (2 features), and medication records (4 features). Detailed information is presented in Table 1.

Due to distinct sampling disparities within the 4-h observation window, features were stratified by temporal granularity. Laboratory parameters, typically collected at 12–24 h intervals, remain relatively static, whereas vital signs from continuous monitoring exhibit dynamic fluctuations. Consequently, seven variables—systolic, diastolic, and mean blood pressure, heart rate, temperature, SpO_2_, and respiratory rate—were designated as high-frequency time series. The remaining 34 features were classified as low-frequency data.

### 2.6. Model Development

#### 2.6.1. MODS Prediction Model

LSTM processes sequential data recursively, with each output depending on the previous state. It performs well in time series prediction tasks, particularly when dealing with short time series [27,28,29,30]. Given the fixed 4-h observation window in this study, LSTM can effectively capture the trends in physiological changes within this short timeframe, making it the preferred modeling algorithm for high-frequency time series data. In contrast, the MLP has simple architecture. It models the nonlinear relationships between features and is well-suited for handling non-temporal structured data. Therefore, MLP is employed to model the low-frequency data [31].

In the pre-trained model for mortality prediction, high-frequency time series data is input into the LSTM module to capture dynamic changes, while low-frequency data is processed by MLP for feature extraction. The outputs from these two pathways are concatenated to form intermediate features, which are then passed through a fully connected layer (FC Layer). These features undergo batch normalization before being passed through a sigmoid activation function to predict the patient’s mortality risk within the next 30 days (“Pre-train Model” section of Figure 2).

After building the pre-trained model, all of its parameters were transferred to the MODS prediction model to serve as initialization weights. Building upon the pre-trained structure, several FC Layers were added to the MODS prediction model to enhance its ability to capture features. The model was then fine-tuned using 80% of the trauma sepsis patient data from the MIMIC-IV database, resulting in the final MODS prediction model (“MODS Model” section of Figure 2).

#### 2.6.2. Five Derivative Models

To assess the contribution of each individual component, we developed five ablation models based on the original model (Detailed parameters are provided in Supplementary Text S1). The variations across these models are characterized by differences in (i) the pre-training dataset, (ii) the use of high-frequency and low-frequency data separation, and (iii) the network backbone.

PT-MLP·LSTM-eICU: Pre-trained on the merged MIMIC-IV and eICU cohort; utilizes high-frequency and low-frequency data separation.

MLP·LSTM: Trained from scratch on 2802 trauma sepsis patients from MIMIC-IV; high-frequency and low-frequency separation enabled.

PT-MLP-eICU: Pre-trained; no frequency separation. The MLP takes 34 low-frequency features plus the 7 high-frequency variables at the end of the time window.

PT-LSTM-eICU: Same input setting as PT-MLP-eICU, but using an LSTM backbone. Low-frequency features are interpolated to 4-h bins (held constant within each bin) and concatenated with high-frequency sequences from the same bin.

PT-MLP·LSTM: Utilizes high-frequency and low-frequency data separation and pre-training on MIMIC-IV data only (49,648 ICU patients), without including the eICU dataset.

### 2.7. Time Windows and Labeling

The model employs a 4h observation window and predicts the occurrence of MODS within the next 6, 12, and 24h. Specifically, the model only receives clinical data observed in the interval $\left(t_{k}-4\;h,\;t_{k}\right]$ as input and predicts whether MODS will occur in the future interval $\left(t_{k},\;t_{k}+h\right]$, where h∈{6h,12h,24h} (“Time window” section of Figure 2). We define $T_{\mathrm{MODS}}$ as the first time point at which MODS occurs, when SOFA scores reach ≥2 in at least two organ systems. For each evaluation time $t_{k}$, we construct a three-dimensional label vector $\left(y_{6}\left(t_{k}\right),\;y_{12}\left(t_{k}\right),\;y_{24}\left(t_{k}\right)\right)$. For each horizon *h*, we set $y_{h}\left(t_{k}\right)=1$ if $t_{k}<T_{\mathrm{MODS}}\leq t_{k}+h$; otherwise, $y_{h}\left(t_{k}\right)=0$ if $T_{\mathrm{MODS}}>t_{k}+h$ or if MODS does not occur within the available follow-up. To mitigate label leakage, windows with $T_{\mathrm{MODS}}\leq t_{k}$ (MODS already present at the evaluation time) are excluded from training and evaluation.

### 2.8. Performance Evaluation

This study used the remaining 20% of trauma sepsis patient data from MIMIC-IV as an internal test set for validation. Additionally, trauma sepsis patient data meeting the inclusion criteria in the eICU database were used for multicenter external validation to assess the model’s generalization ability.

The model’s performance was primarily evaluated using the area under the curve (AUC), while the accuracy (ACC), sensitivity (TPR) and specificity (TNR) were calculated to comprehensively compare the performance of different models in the MODS prediction task (“Validation” section of Figure 2). The calculation formulas for each evaluation indicator are provided in Supplementary Table S1.

To quantify uncertainty, we constructed 95% confidence intervals (CIs) using a patient-level bootstrap procedure (B = 2000 resamples) to account for within-patient correlation. In each resample, we sampled patients with replacement (with the number of sampled patients equal to that in the original cohort), assigned each record a weight corresponding to the number of times its patient was selected, and recalculated the AUC (using sample weights) as well as ACC/TPR/TNR. The 2.5th and 97.5th percentiles of the resulting bootstrap distributions were taken as the bounds of the 95% CIs.

### 2.9. Model Interpretation

The SHAP (SHapley Additive exPlanations) method offers a theoretically rigorous, visually intuitive, and quantifiable way to explain the contributions of features in complex models [32]. In this study, we used SHAP to assess the importance of input features and randomly selected positive and negative samples for typical example analysis to further illustrate the model’s reasoning process in real-world predictions (“Interpretation” section of Figure 2).

### 2.10. Model Parameters

We used a 4-h observation window and formulated MODS risk prediction as a multi-label classification task, predicting MODS onset within the subsequent 6, 12, and 24 h, consistent with the overall pipeline described in the manuscript. For reproducibility, we fixed the random seed to 42 (NumPy v1.24.3 and PyTorch v2.3.0) and enabled deterministic CuDNN behavior to reduce run-to-run variability. Model parameters were optimized using AdamW with a learning rate of $1\times 10^{-5}$ and weight decay of $1\times 10^{-3}$, using a batch size of 4096. Training proceeded for up to 300 epochs with early stopping (patience = 30): training was terminated if the mean validation AUC (averaged across the 6/12/24-h outputs) did not improve for 30 consecutive epochs, and the checkpoint achieving the best mean validation AUC was retained for final evaluation. The training objective was the average binary cross-entropy over the three outputs, implemented with BCEWithLogitsLoss:

(2) $$L=\frac{1}{N}\sum_{i=1}^{N}\frac{1}{3}\sum_{k\in \{6,12,24\}}\mathrm{BCEWithLogits}\;\left(z_{i,k},\;y_{i,k}\right)$$

where $z_{i,k}\in R$ denotes the model logits for sample *i*, $y_{i,k}\in \left\{0,1\right\}$ denotes the corresponding ground-truth labels for the three prediction horizons (6h/12h/24h), and *N* is the batch size.

Supplementary Table S4 presents the detailed architecture and hyperparameters of the model.

## 3. Results

### 3.1. Development Cohort Analysis

According to the inclusion criteria shown in Figure 1, 3502 trauma sepsis patients were included from the MIMIC-IV database, of whom 1854 (52.94%) developed MODS and 1648 did not. Table 2 summarizes the baseline characteristics of these patients, the baseline characteristics of eICU patients are presented in Supplementary Table S2. Compared with non-MODS patients, those with MODS had an average ICU stay that was 5 days longer and an in-hospital mortality rate nearly 20% higher, both differences being statistically significant (*p* < 0.001). These findings suggest that MODS significantly impacts the clinical prognosis of trauma sepsis patients.

### 3.2. Ablation Experiment

#### 3.2.1. Model Performance Under Different Prediction Windows

**Figure 3:**
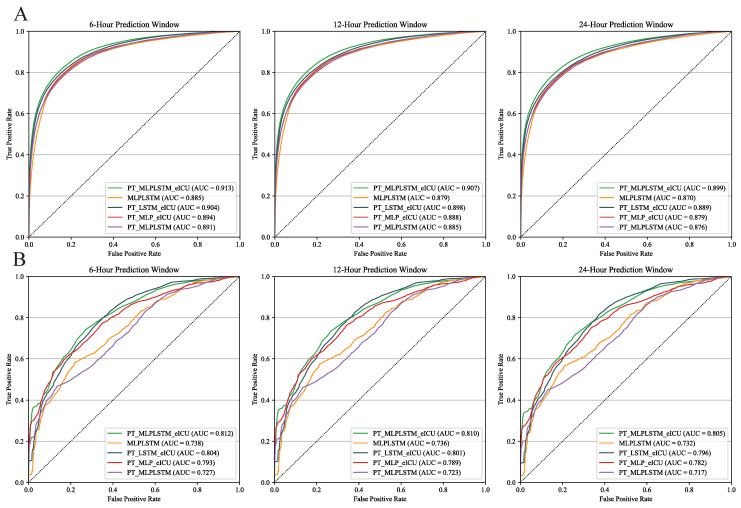
Receiver operating characteristic (ROC) curves of each model at 6-, 12-, and 24-h prediction windows. (**A**) Internal validation using the MIMIC-IV; (**B**) Multicenter external validation using the eICU database.

Table 3 presents the performance of the five models in the internal validation of MIMIC-IV, detailed performance of the external validation on the eICU dataset is presented in Supplementary Table S3. Figure 3 compares the ROC curves of each model under different prediction windows. As the prediction window lengthens, the performance of each model decreases slightly but remains relatively stable. For instance, the internal validation AUC of PT-MLP·LSTM-eICU under the 24-h prediction window is 0.899, and the external validation AUC is 0.805, representing only 0.014 and 0.007 reductions compared to the 6-h window, respectively. Notably, PT-MLP·LSTM-eICU consistently achieves the best performance across the 6, 12, and 24-h prediction windows. Its 24-h AUC (0.8991) even surpasses the 6-h AUC of the MLP·LSTM model (0.8846).

#### 3.2.2. Comparative Analysis of Models

Table 4 highlights the impact of each model component on overall performance. PT-MLP·LSTM-eICU achieved the best performance in both MIMIC-IV internal validation and eICU external validation, with average AUCs of 0.906 and 0.809 across the three prediction windows, respectively. In contrast, the average AUC of the non-pretrained MLP-LSTM model decreased by 0.028 and 0.074, respectively.

Compared to the best model, PT-LSTM-eICU showed a decrease in performance, with AUCs of 0.897 in MIMIC-IV and 0.800 in eICU. This suggests that the high- and low-frequency data separation strategy can improve the generalization performance of the model. When PT-MLP-eICU replaced LSTM with MLP, the internal validation AUC dropped to 0.887, underscoring LSTM’s advantage in processing time-series data.

Finally, in the eICU external validation, the AUC of PT-MLP·LSTM decreased significantly to 0.722, while it remained at 0.884 in MIMIC-IV. This suggests that pre-training using only MIMIC-IV data improves internal performance but still has limited external generalization capability.

#### 3.2.3. Comparison with SOFA

Table 5 presents a performance comparison of different models on the MIMIC and eICU datasets. The experimental results indicate that the proposed full-parameter model, ALL-var, achieves the best performance across all evaluation metrics. Specifically, the AUC of SOFA-var on the MIMIC dataset is approximately 14% higher than that of SOFA-only. Moreover, on both datasets, SOFA-var consistently outperforms noSOFA-var (e.g., on the MIMIC dataset, the AUC value for the 6-h prediction window of SOFA-var is 0.880, while that of noSOFA-var is 0.809.), while ALL-var further surpasses all models that use only a single subset of variables.

### 3.3. Relationship Between Model Performance and Dataset Size

**Figure 4:**
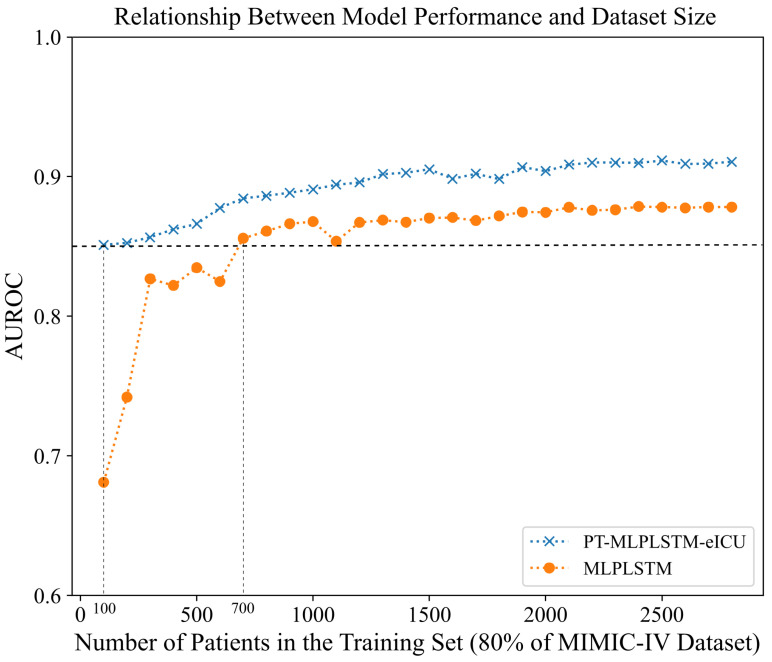
The relationship between model performance and dataset size.

Figure 4 shows the performance changes in PT-MLP·LSTM-eICU and MLP·LSTM under different training data volumes. Building upon the completion of the 30-day mortality pre-training task, PT-MLP·LSTM-eICU gradually expanded the size of the fine-tuning dataset to assess model performance under different data volumes. Meanwhile, MLP·LSTM adjusted the size of its training set for comparison. We progressively expanded the size of the training data by adding 100 patients at a time and calculated the average AUCs for the three prediction time windows on the same test set.

The results show that when the training data volume was only 100 cases (3.57% of the total samples), PT-MLP·LSTM-eICU already achieved an AUC of 0.846, whereas MLP·LSTM reached only 0.681, resulting in a performance gap of 24.23%. As the sample size increased, the AUCs of both models improved, but the improvement for MLP·LSTM was more pronounced. For instance, at 300 training samples, MLP·LSTM’s AUC rose to 0.827 (an increase of 21.44%).

The horizontal dotted line in Figure 4 indicates the performance baseline of PT-MLP·LSTM-eICU under 100 training samples. MLP·LSTM required about 700 training samples to reach a comparable AUC, highlighting its greater reliance on large training datasets. Ultimately, when all available data were used, the AUC gap narrowed to 0.028 (from an initial 0.165), reducing the relative performance gap from 24.23% to 3.09%.

### 3.4. Model Calibration

**Figure 5:**
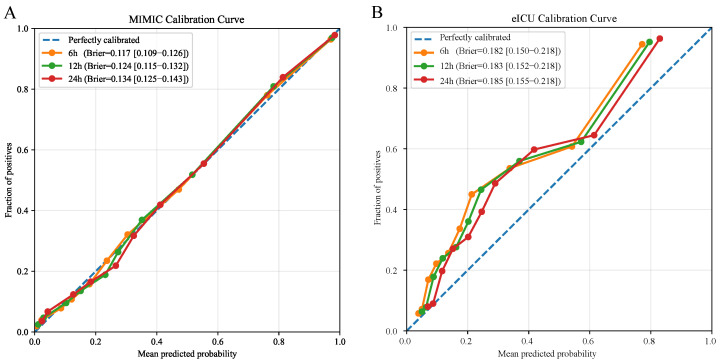
Calibration curves with corresponding Brier scores. (**A**) Internal validation cohort; (**B**) External validation cohort; The dashed line indicates perfect calibration.

In the internal validation cohort (Figure 5A), calibration curves for the 6 h, 12 h, and 24 h windows closely followed the 45° reference line with only minor deviations in the low-to-intermediate risk range. The Brier scores were 0.117 [0.109, 0.126], 0.124 [0.115, 0.132], and 0.134 [0.125, 0.143], respectively, indicating a modest increase in overall prediction error with longer horizons. In the external validation cohort (Figure 5B), calibration deviated more substantially from the reference line, with curves predominantly above the diagonal, suggesting systematic risk underestimation. The Brier scores were 0.182 [0.150–0.218], 0.183 [0.152–0.218], and 0.185 [0.155–0.218], higher than those in internal validation.

### 3.5. Clinical Threshold Performance

Sensitivity and specificity at the predefined risk thresholds (10%, 20%, and 30%) are summarized in Table 6. Overall, both cohorts exhibited a consistent pattern whereby increasing the threshold decreased sensitivity while increasing specificity. In the internal validation cohort (MIMIC), the 10% threshold achieved the highest sensitivity but relatively low specificity; raising the threshold to 20% substantially improved specificity and yielded a more balanced trade-off. A similar trend was observed in the external validation cohort (eICU); however, at comparable thresholds, specificity was generally lower than that in MIMIC.

### 3.6. Model Interpretation

#### 3.6.1. Feature Contributions

**Figure 6:**
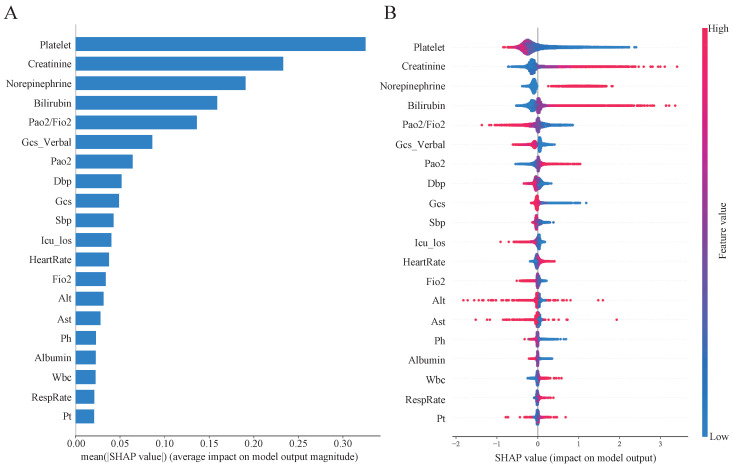
Interpretability analysis based on SHAP algorithm. (**A**) Analysis of the weights of the 20 most important features; (**B**) The impact of the 20 most important features on the model results, where the horizontal axis represents the relative relationship between the feature value and the average value of the feature. Blue indicates a reduced MODS risk, and red indicates an increased MODS risk.

Based on the SHAP algorithm, we conducted an explanatory analysis of the PT-MLP·LSTM-eICU model under a 6-h prediction window. Figure 6 shows the top 20 features and their weights that have the greatest impact on model prediction. Platelet count is the most critical predictor variable, and a decrease in its number will significantly increase the risk of MODS. Secondly, increased creatinine and increased norepinephrine dose are closely related to the occurrence of MODS (Figure 6B). The three most important features are Platelet, Creatinine, and Norepinephrine, respectively.

Supplementary Figure S1 presents the hourly mean absolute SHAP values, mean of absolute SHAP, for high-frequency time series data within the four-hour observation window. Overall, blood pressure related features exhibit the greatest contribution. DBP is the most influential feature across all hours and shows mild temporal heterogeneity. Its contribution increases progressively from Hour 1, peaks at Hour 3, and then decreases slightly at Hour 4, which is closest to the prediction time point, while remaining the top contributor.

#### 3.6.2. Case Analysis

**Figure 7:**
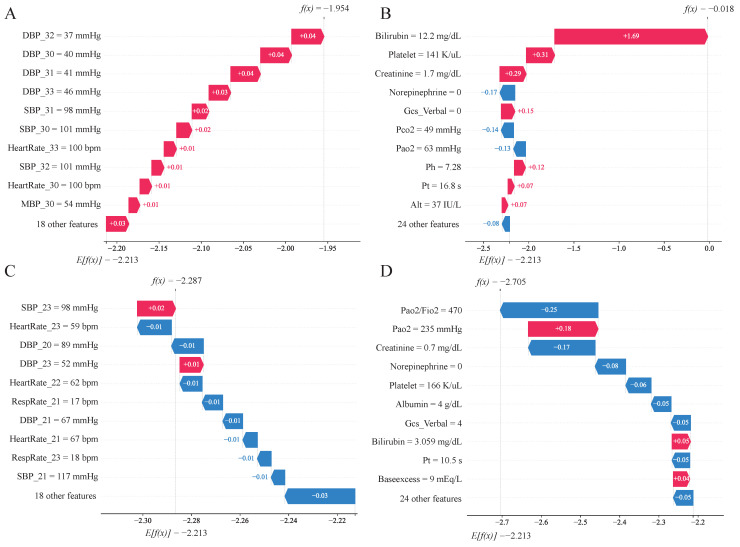
Contribution of individual factors. (**A**,**B**) A MODS patient, where (**A**) shows high-frequency time series variables and (**B**) shows low-frequency variables; (**C**,**D**) A non-MODS patient, where (**C**) shows high-frequency time series variables and (**D**) shows low-frequency variables; E[f(X)], base value, which is the output of the model without any feature information; f(x), the model output of this sample, it equals the base value plus the sum of the SHAP values for all features; DBP_*n*_, DBP at the *n*-th hour after ICU admission, and other high-frequency time-series variables follow the same format. The horizontal axis represents the feature contribution values (SHAP values), while the vertical axis lists the most influential features from top to bottom. Each arrow represents how a specific feature increases (red) or decreases (blue) the patients’ risk for MODS.

We randomly selected one MODS patient and one non-MODS patient, and performed a typical analysis based on a 6-h prediction window. The results are shown in Figure 7.

The MODS patient was a 52-year-old male. The data collection time was from the 30th to the 33rd hour of admission. The model prediction time window was from the 34th to the 39th hour. MODS was finally diagnosed at the 37th hour. SHAP analysis showed that the patient had multiple high-risk features: among the high-frequency time series variables, both systolic and diastolic blood pressure were low (Figure 7A); among the low-frequency variables, total bilirubin was as high as 12.2 mg/dL, which was the most contributing indicator, followed by low platelet count (141 K/uL) and elevated creatinine (Figure 7B). These abnormal indicators have higher SHAP weights in the model, directly contributing to its high-risk predictions for MODS.

The non-MODS patient was an 86-year-old female. The analysis window was from the 20th to the 23rd hour of admission. MODS did not develop in the end. Model feature analysis showed that most of the patient’s indicators (such as oxygenation index, creatinine, lactate, heart rate) were within the normal range (Figure 7C,D). Although the high PaO_2_ and low SBP at the 23rd hour slightly increased the risk assessment, the overall risk (f(x)) was still below the threshold (E[f(X)]), and the model predicted that MODS would not occur in the next 6 h, which was consistent with the actual results.

## 4. Discussion

Due to the complex mechanism and high mortality rate of MODS, it is a common and critical complication in trauma sepsis patients. Real-time prediction of MODS for this population has significant clinical value. Currently, existing studies have mainly focused on ordinary ICU patients, with a lack of special research on trauma sepsis patients. This study helps to address that gap. Methodologically, we introduced pre-training and high- and low-frequency data separation strategies to improve the prediction performance of the model. We also proved that pre-training was more advantageous under small samples.

A recent trauma-induced sepsis study used variables from the first 24 h of ICU admission to develop a nomogram and several machine-learning models, and reported an AUC of 0.769 in temporal validation for a random forest model [33]. However, the study was based on a single-center dataset and did not include external validation, which may limit its generalizability. In contrast, our model updates in real time using 4-h rolling windows and provides 6/12/24-h forecasts, achieving AUCs of 0.913/0.907/0.899 (Table 3) in internal validation and 0.812/0.810/0.805 in external eICU validation. These results suggest that combining pre-training with dynamic time-series modeling may improve early warning performance in trauma sepsis and support clinical decision-making across actionable time horizons.

The pre-training transfer model we developed uses the prediction of the patients’ mortality rate in the next 30 days as the pre-training task. As shown in Table 2, the mortality rate of MODS patients is significantly higher than that of non-MODS patients. Therefore, mortality prediction as a pre-training target has strong clinical relevance. The ablation experiment results (Table 3 and Table 4) further show that pre-training significantly improves the AUC performance of the model in internal and external validation. In particular, Figure 4 illustrates that the advantages of pre-training are particularly prominent in small sample tasks. When only 100 patients are used for training, the AUC of the non-pre-trained model is only 0.681, while the pre-trained model can reach 0.846, an improvement of 24.22%. We believe that the effectiveness of the pre-training strategy lies in the fact that the model learns clinically critical features, such as vital sign changes and laboratory indicators, from over 200,000 ICU patients during the mortality prediction task. These features are highly universal and provide valuable prior knowledge support for MODS prediction, thereby improving the model performance and transferability.

In addition, incorporating eICU data into the pre-training stage significantly improved not only the performance in multi-center external validation, but also the internal validation performance. This shows that by incorporating multi-source data, the pre-training process can be exposed to more diverse patient characteristics, which can enhance the generalization ability and robustness of the model. This result also suggests that more diverse data should be further incorporated into the future pre-training process to improve the cross-center adaptability and overall performance of the model.

Another advantage of the model is its introduction of multi-label classification, which can simultaneously predict the occurrence of MODS in multiple future time windows (6 h, 12 h and 24 h). In internal validation, the AUCs corresponding to the three prediction time windows were 0.913 (95% CI: 0.907, 0.920), 0.907 (95% CI: 0.901, 0.914) and 0.899 (95% CI: 0.891, 0.906), respectively. The results show that the performance of the model decreases slightly with the extension of the prediction time window, and the overall decrease is only 0.0135, which still maintains a high prediction accuracy. This staged risk prediction provides clinicians with risk assessment at different time scales, so that different prediction windows can be selected according to clinical practice to improve the comprehensiveness of decision-making.

In view of the significant difference in the acquisition frequency between high-frequency time series variables and low-frequency variables in the 4-h observation window, we introduced a differentiated processing strategy. High-frequency time series variables were input into an LSTM network, which excels at capturing temporal dependencies, while low-frequency variables were input into an MLP, known for its simple structure and efficient computation. In the ablation experiment, the PT-MLPLSTM-eICU model had an average AUC of 0.906 in internal validation and 0.809 in external validation. In contrast, the AUCs of PT-LSTM-eICU and PT-MLP-eICU, which did not use this strategy, were lower, verifying the effectiveness of this method. We believe that using different substructures to process various types of variables and analyzing patient characteristics from multiple perspectives enables the model to learn richer and more diverse feature representations. Compared to models that did not adopt this strategy, this strategy exhibits stronger representation and better generalization capabilities when faced with diverse inputs, thereby significantly enhancing the model’s generalization performance.

In comparison with the traditional SOFA-based framework, our experiments further support the effectiveness of PT-MLPLSTM-eICU. First, the model built on SOFA-related variables outperformed prediction using the SOFA score alone, suggesting that—relative to the discretized SOFA score—directly modeling the underlying continuous SOFA-related physiological measurements reduces information loss and captures richer clinical signals. Second, it also outperformed models constructed using SOFA-unrelated variables, highlighting the central role of organ dysfunction indicators encompassed by SOFA in reflecting disease severity. Nevertheless, PT-MLPLSTM-eICU achieved the best overall performance, indicating that SOFA-related and non-SOFA variables provide complementary information for predicting outcomes in critically ill patients.

Beyond discrimination, we further examined model calibration and clinical trade-offs under different risk thresholds. In internal validation, overall calibration was robust as the calibration curves closely followed the reference line. Furthermore, Brier scores of 0.117, 0.124, and 0.134 suggested that extending the prediction horizon led to a modest increase in error. In contrast, calibration was notably poorer in external validation, with curves lying predominantly above the diagonal to indicate a systematic underestimation of risk. Correspondingly, the Brier score increased to 0.182–0.185, implying that cross-center distribution shifts may compromise the transportability of predicted probabilities. Consistent with this, both cohorts exhibited the expected pattern across 10%, 20%, and 30% thresholds where higher thresholds decreased sensitivity while increasing specificity. For instance, a 10% threshold in MIMIC favored screening, whereas 20% provided a more balanced trade-off. However, at the same thresholds, eICU showed consistently lower specificity. This suggests a higher false-positive burden and indicates that the direct transfer of fixed thresholds may be suboptimal. Therefore, in real-world deployment, thresholds should be re-selected according to the target use case. Additionally, local recalibration or incremental updating based on site-specific data should be performed when necessary to improve robustness across centers.

The model developed in this study also has good interpretability. Previous studies have shown that a low platelet count is a key risk factor for MODS. Its impact is not only reflected in the bleeding tendency, but also widely involves microcirculatory disorders, immune dysfunction and imbalanced regulation of inflammatory responses [34,35,36,37]. Increased creatinine is an early warning sign of MODS, indicating that kidney function is impaired and cannot effectively complete the clearance of metabolites, regulate water and electrolyte balance and maintain a stable internal environment, thereby accelerating the progression of MODS through metabolic disorders, inflammatory amplification and organ-to-organ interactions [38]. The injection of norepinephrine may lead to insufficient microcirculatory perfusion of important organs, which in turn causes tissue hypoxia, metabolic disorders and imbalanced inflammatory responses, especially prone to damage the kidneys and mesenteric organs, and eventually lead to MODS [39,40]. Elevated bilirubin suggests sepsis-associated hepatic dysfunction or cholestasis, reflecting an increased inflammatory burden and impaired hepatic microcirculatory perfusion [41]. Hepatic dysfunction compromises the clearance and metabolism of endotoxins and inflammatory mediators, thereby amplifying systemic inflammation and, through liver–kidney and liver–lung organ crosstalk, exacerbating tissue hypoxia and metabolic derangements, ultimately increasing the risk of MODS [42]. Continuously low blood pressure may lead to insufficient organ perfusion, causing cellular hypoxia, metabolic disorders, toxin accumulation and systemic inflammatory responses, and eventually evolving into MODS [43,44].

In addition, in the typical analysis of patients (Figure 7C), it can be observed that SBP at different times within the 4-h observation window has different effects on the model prediction results. For example, the SBP at the 23rd hour is 98, and its SHAP value is +0.02, and the SBP at the 21st hour is 117, and the SHAP value is −0.01. DBP in Figure 7A also shows similar characteristics. This demonstrates that the LSTM module can effectively capture dynamic relationships within high-frequency time series data. It fully considers the contributions of data at different time points to the prediction results, and it overcomes the limitations of traditional static models that can only make judgments based on the characteristics of a single moment.

### Study Limitations

This study has several limitations. Primarily, as a retrospective analysis leveraging two public critical care databases, case identification and variable extraction relied on structured EHR data. These data are susceptible to documentation variability, coding heterogeneity, and potential misclassification, factors that may influence both the estimated incidence of MODS and the model’s predictive performance.

In particular, within the trauma sepsis cohort, a critical constraint was the unavailability of key injury-specific descriptors, such as the injury mechanism, anatomic injury distribution, and formal severity scores (e.g., ISS/AIS). Consequently, these variables were excluded from model training, which may obscure heterogeneity in performance across different trauma subtypes.

Furthermore, our model did not explicitly include pre-existing comorbidities as predictors; therefore, residual confounding by baseline health status cannot be fully excluded. Future work will incorporate standardized comorbidity indices and additional longitudinal history to further evaluate robustness.

Methodologically, substantial data harmonization was required to address discrepancies across databases, including differences in measurement units, sampling frequencies, and missingness patterns. The reliance on mean or extreme value imputation to address missing values may introduce bias and fails to capture imputation uncertainty; utilizing more sophisticated imputation strategies could further improve model robustness.

Regarding the pre-training task, this study focused exclusively on “30-day mortality prediction”, without encompassing other adverse prognostic indicators for MODS patients.

Lastly, the external validation cohort from the eICU database was relatively small (*n* = 110), and prospective validation within real-world clinical workflows was not performed. Validation in larger multicenter cohorts and prospective deployment studies is necessary before routine clinical adoption can be recommended.

## 5. Conclusions

In this study, we developed a real-time prediction model for MODS in trauma sepsis patients by leveraging pre-training on the MIMIC-IV and eICU databases. We conducted a comprehensive evaluation of the model’s performance, assessing the impact of pre-training data diversity, training set size, the integration of high- and low-frequency input features, and varying prediction windows. Exhibiting robust interpretability, generalizability, and portability, the proposed model demonstrates significant potential for clinical translation. Future directions include prospective validation in larger, diverse multicenter cohorts and the optimization of the model through richer, disease-relevant pre-training and multimodal data integration. Ultimately, this model is poised to empower medical staff with effective support for early risk stratification and intervention in clinical settings.

## Figures and Tables

**Table 1:** Model input features.

Features	Details
Demographics	Age, Gender, ICU LOS
Vital Signs	Heart rate *, Mean Blood Pressure (MBP) *, Systolic Blood Pressure (SBP) *, Diastolic Blood Pressure (DBP) *, Temperature *, SpO_2_ *, Respiratory Rate *, GCS, GCS Motor, GCS Verbal, GCS Eyes
Biochemical Markers	PaO_2_, Hematocrit, WBC, Creatinine, BUN, Sodium, Albumin, Bilirubin, Glucose, pH, PaCO_2_, PaO_2_/FiO_2_, Platelet, PT, Potassium, ALT, AST, Base Excess, Chloride, Total CO_2_, Lactate, Free Calcium, FiO_2_
Medication Records	Epinephrine, Norepinephrine, Dopamine, Dobutamine

* High-frequency time-series data.

**Table 2:** Baseline characteristics of trauma sepsis patients in MIMIC-IV.

Variables	Overall (3502)	MODS (1854)	Non-MODS (1648)	*p* Value
Age (mean ± std in years)	64.79 ± 19.73	64.97 ± 18.51	64.58 ± 21.02	0.557
Gender (males)	2237 (63.88%)	1251 (67.48%)	986 (59.83%)	<0.001
ICU length of stay (days)	6.04 ± 5.95	8.16 ± 6.68	3.65 ± 3.79	<0.001
Hospital length of stay (days)	15.61 ± 18.66	19.21 ± 22.73	11.55 ± 11.29	<0.001
ICU type (%)				<0.001
CCU	230 (6.57%)	155 (8.36%)	75 (4.55%)	
SICU	532 (15.19%)	250 (13.48%)	282 (17.11%)	
Neuro Stepdown	36 (1.03%)	7 (0.38%)	29 (1.76%)	
Neuro Intermediate	55 (1.57%)	7 (0.38%)	48 (2.91%)	
CVICU	150 (4.28%)	96 (5.18%)	54 (3.28%)	
Neuro SICU	126 (3.6%)	59 (3.18%)	67 (4.07%)	
TSICU	1389 (39.66%)	718 (38.73%)	671 (40.72%)	
MICU	686 (19.59%)	400 (21.57%)	286 (17.35%)	
MICU/SICU	298 (8.51%)	162 (8.74%)	136 (8.25%)	
Ethnicity (%)				0.005
Asian	73 (2.08%)	42 (2.27%)	31 (1.88%)	
Black	217 (6.2%)	105 (5.66%)	112 (6.8%)	
Hispanic	119 (3.4%)	64 (3.45%)	55 (3.34%)	
Other/Unknown	800 (22.84%)	468 (25.24%)	332 (20.15%)	
White	2293 (65.48%)	1175 (63.38%)	1118 (67.84%)	
Mortality	711 (20.30%)	541 (29.18%)	170 (10.32%)	<0.001

**Table 3:** Internal validation performance of each model under different prediction time windows.

Model	Window	AUC (95%CI)	ACC (95%CI)	TPR (95%CI)	TNR (95%CI)
PT-MLP·LSTM-eICU	6 h	0.913 (0.907, 0.920)	0.831 (0.818, 0.842)	0.829 (0.814, 0.845)	0.831 (0.810, 0.850)
	12 h	0.907 (0.901, 0.914)	0.823 (0.811, 0.836)	0.829 (0.806, 0.839)	0.820 (0.803, 0.844)
	24 h	0.899 (0.891, 0.906)	0.816 (0.807, 0.825)	0.817 (0.799, 0.828)	0.815 (0.800, 0.830)
MLP·LSTM	6 h	0.884 (0.876, 0.892)	0.810 (0.803, 0.818)	0.812 (0.797, 0.825)	0.809 (0.797, 0.816)
	12 h	0.879 (0.870, 0.887)	0.803 (0.794, 0.809)	0.806 (0.795, 0.817)	0.801 (0.790, 0.809)
	24 h	0.870 (0.860, 0.879)	0.795 (0.785, 0.802)	0.794 (0.783, 0.801)	0.796 (0.784, 0.807)
PT-LSTM-eICU	6 h	0.904 (0.897, 0.911)	0.824 (0.812, 0.830)	0.815 (0.804, 0.824)	0.829 (0.820, 0.839)
	12 h	0.898 (0.890, 0.905)	0.813 (0.804, 0.822)	0.813 (0.803, 0.824)	0.814 (0.803, 0.823)
	24 h	0.889 (0.879, 0.897)	0.804 (0.792, 0.81)	0.800 (0.785, 0.816)	0.807 (0.794, 0.817)
PT-MLP-eICU	6 h	0.894 (0.886, 0.901)	0.811 (0.804, 0.823)	0.819 (0.802, 0.824)	0.808 (0.802, 0.825)
	12 h	0.888 (0.879, 0.895)	0.810 (0.798, 0.817)	0.803 (0.796, 0.818)	0.813 (0.796, 0.819)
	24 h	0.879 (0.870, 0.887)	0.801 (0.788, 0.808)	0.793 (0.786, 0.810)	0.805 (0.786, 0.811)
PT-MLP·LSTM	6 h	0.891 (0.883, 0.898)	0.809 (0.786, 0.826)	0.806 (0.776, 0.819)	0.811 (0.767, 0.844)
	12 h	0.885 (0.876, 0.893)	0.802 (0.783, 0.818)	0.802 (0.776, 0.823)	0.802 (0.765, 0.821)
	24 h	0.876 (0.867, 0.885)	0.794 (0.779, 0.804)	0.792 (0.774, 0.837)	0.795 (0.766, 0.836)

**Table 4:** Impact of each model component on performance.

Model	Pre-Train	MLP	LSTM	eICU	MIMIC-IV AUC (Mean)	eICU AUC (Mean)
PT-MLP·LSTM-eICU	✓	✓	✓	✓	0.906	0.809
MLP·LSTM		✓	✓		0.878	0.735
PT-LSTM-eICU	✓		✓	✓	0.897	0.800
PT-MLP-eICU	✓	✓		✓	0.887	0.788
PT-MLP·LSTM	✓	✓	✓		0.884	0.722

PT, Pre-training; MLP·LSTM, the model is optimized for high-frequency and low-frequency data separation; LSTM, all physiological parameters are uniformly input into the LSTM; MLP, all physiological parameters are consistently input into the MLP; eICU, the pre-training dataset incorporates eICU data; AUC, the average AUC of the model in three prediction windows; Checkmark, the model is represented as having this partial structure.

**Table 5:** Comparison with SOFA.

		6-h Prediction Window		12-h Prediction Window		24-h Prediction Window	
Dataset	Method	AUC	ACC	AUC	ACC	AUC	ACC
MIMIC-IV	SOFA-only	0.748 (0.700, 0.789)	0.734 (0.675, 0.787)	0.736 (0.685, 0.783)	0.725 (0.670, 0.776)	0.727 (0.675, 0.777)	0.711 (0.660, 0.758)
	SOFA-var	0.880 (0.863, 0.895)	0.806 (0.788, 0.823)	0.869 (0.851, 0.886)	0.795 (0.776, 0.813)	0.854 (0.834, 0.873)	0.777 (0.760, 0.799)
	noSOFA-var	0.809 (0.782, 0.833)	0.730 (0.705, 0.755)	0.805 (0.778, 0.829)	0.728 (0.701, 0.748)	0.800 (0.772, 0.824)	0.715 (0.692, 0.738)
	ALL-var	0.913 (0.907, 0.920)	0.831 (0.818, 0.842)	0.907 (0.901, 0.914)	0.823 (0.811, 0.836)	0.899 (0.891, 0.906)	0.816 (0.807, 0.825)
eICU	SOFA-only	0.730 (0.652, 0.816)	0.730 (0.646, 0.810)	0.722 (0.628, 0.820)	0.732 (0.653, 0.811)	0.681 (0.559, 0.797)	0.722 (0.636, 0.805)
	SOFA-var	0.771 (0.716, 0.822)	0.680 (0.619, 0.735)	0.764 (0.711, 0.819)	0.666 (0.605, 0.723)	0.759 (0.700, 0.813)	0.642 (0.572, 0.703)
	noSOFA-var	0.756 (0.702, 0.809)	0.642 (0.582, 0.699)	0.749 (0.695, 0.803)	0.633 (0.574, 0.69)	0.742 (0.688, 0.794)	0.630 (0.574, 0.685)
	ALL-var	0.812 (0.757, 0.864)	0.735 (0.681, 0.784)	0.810 (0.754, 0.862)	0.734 (0.679, 0.782)	0.805 (0.748, 0.857)	0.724 (0.673, 0.777)

SOFA-only: A logistic regression model using SOFA score only as input; SOFA-var: Built on the paper’s best model (PT-MLP·LSTM-eICU), but using only SOFA-related variables as features; noSOFA-var: Also based on PT-MLP·LSTM-eICU, but using only non-SOFA-related variables as features; ALL-var: Identical to PT-MLP·LSTM-eICU, with all features included.

**Table 6:** Sensitivity and specificity at predefined clinical risk thresholds.

		6-h Prediction Window		12-h Prediction Window		24-h Prediction Window	
Dataset	Threshold	Sensitivity	Specificity	Sensitivity	Specificity	Sensitivity	Specificity
MIMIC-IV	10%	0.909 (0.899, 0.919)	0.676 (0.660, 0.691)	0.948 (0.941, 0.955)	0.523 (0.506, 0.540)	0.967 (0.962, 0.972)	0.376 (0.358, 0.393)
	20%	0.630 (0.605, 0.656)	0.956 (0.950, 0.961)	0.726 (0.705, 0.748)	0.898 (0.890, 0.906)	0.816 (0.799, 0.832)	0.791 (0.779, 0.803)
	30%	0.411 (0.380, 0.442)	0.991 (0.989, 0.993)	0.431 (0.401, 0.461)	0.990 (0.988, 0.992)	0.478 (0.449, 0.507)	0.985 (0.982, 0.997)
eICU	10%	0.881 (0.825, 0.931)	0.494 (0.390, 0.596)	0.913 (0.862, 0.958)	0.439 (0.335, 0.546)	0.958 (0.923, 0.987)	0.304 (0.204, 0.415)
	20%	0.666 (0.564, 0.764)	0.785 (0.705, 0.856)	0.748 (0.665, 0.830)	0.714 (0.618, 0.796)	0.807 (0.738, 0.871)	0.614 (0.511, 0.706)
	30%	0.533 (0.421, 0.644)	0.879 (0.810, 0.932)	0.550 (0.443, 0.656)	0.970 (0.800, 0.925)	0.579 (0.475, 0.683)	0.844 (0.768, 0.906)

## Data Availability

The original data presented in the study are openly available in GitHub at https://github.com/subcqfq/Sepsis_MODS/ (accessed on 5 January 2026).

## Supplementary Materials

The following supporting information can be downloaded at: https://www.mdpi.com/article/10.3390/diagnostics16020270/s1, Table S1: Formulas and descriptions of each evaluation indicator. Table S2: Baseline characteristics of trauma sepsis patients in eICU. Table S3: External validation performance of each model under different prediction time windows. Table S4. Architecture and hyperparameters. Text S1: Model parameter settings. Figure S1: Temporal contribution heatmap.

## Abbreviations

The following abbreviations are used in this manuscript:

MODS	Multiple Organ Dysfunction Syndrome
AUC	Area Under the Curve
ACC	Accuracy
SHAP	SHapley Additive exPlanations
CI	Confidence Interval
PaO_2_	Partial pressure of arterial oxygen
PaCO_2_	Partial pressure of arterial carbon dioxide
FiO_2_	Fraction of inspired oxygen
PaO_2_/FiO_2_	Oxygenation index
GCS	Glasgow Coma Scale
SBP	Systolic blood pressure
DBP	Diastolic blood pressure
ICU LOS	ICU length of stay
WBC	White blood cell count
PT	Prothrombin time
ALT	Alanine aminotransferase
AST	Aspartate aminotransferase
