# Supplementary material for: A Real-Time Dynamic Warning Method for MODS in Trauma Sepsis Patients Based on a Pre-Trained Transfer Learning Algorithm
Source: Diagnostics (Basel). 2026 Jan 14;16(2):270. doi: 10.3390/diagnostics16020270 (PMC12840517; doi:10.3390/diagnostics16020270)
Supplement: Supplementary file 1 [file diagnostics-16-00270-s001.zip › diagnostics-3993260-supplementary.pdf]

Supplementary material online for

"A Real-Time Dynamic Warning Method for MODS in Trauma Sepsis Patients Based on a Pre-trained Transfer Learning Algorithm"

This supplementary material includes:

Supplementary Table S1: Formulas and descriptions of each evaluation indicator

Supplementary Table S2: Baseline characteristics of trauma sepsis patients in eICU

Supplementary Table S3: External validation performance of each model under different prediction time windows

Supplementary Table S4. Architecture and hyperparameters

Supplementary Text S1: Model parameter settings

Supplementary Figure S1: Temporal contribution heatmap

Supplementary Table S1. Formulas and descriptions of each evaluation indicator.

Indicator	Formula	Description
ACC	$Accuracy = \frac{TP+TN}{TP+TN+FP+FN}$	Probability of making a correct prediction
AUC	$AUC = \frac{\sum_{d=1}^{100}(TPR_d + TPR_{d-1})(FPR_d + FPR_{d-1})}{2}$	Area under the subject's work characteristic curve
TPR	$TPR = \frac{TP}{TP+FN}$	Proportion of positive samples correctly predicted to be in the positive category
TNR	$TNR = \frac{TN}{TN+FP}$	Proportion of negative samples correctly predicted to be in the negative category

Supplementary Table S2. Baseline characteristics of trauma sepsis patients in eICU.

	Overall (110)	MODS (88)	non-MODS (22)	P value
Age (mean ± std in years)	60.84±17.79	61.68±16.90	57.57±20.99	0.401
Gender (males)	71 (64.55%)	58 (65.91%)	13 (59.09%)	0.727
ICU length of stay (days)	9.91±10.06	11.29±10.47	4.51±5.99	<0.001
Hospital length of stay (days)	15.51±13.20	16.60±13.57	8.79±9.57	<0.001
Ethnicity (%)				0.338
Asian	4 (3.64%)	2 (2.27%)	2 (9.1%)	
African American	4 (3.64%)	4 (4.55%)	0 (0%)	
Caucasian	88 (80%)	70 (79.55%)	18 (81.82%)	

Hispanic	9 (8.18%)	7 (7.95%)	2 (9.1%)	
Other/Unknown	5 (4.55%)	5 (5.68%)	0 (0%)	
Mortality	24 (21.82%)	22 (25%)	2 (9.09%)	<0.001

Supplementary Table S3. External validation performance of each model under different prediction time windows.

Model	AUC	ACC	TPR	TNR
PT-MLP･LSTM-eICU				
6 hours	0.812 (0.757, 0.864)	0.735 (0.681, 0.784)	0.738 (0.678, 0.787)	0.732 (0.679, 0.786)
12 hours	0.810 (0.754, 0.862)	0.734 (0.679, 0.782)	0.723 (0.676, 0.784)	0.741 (0.676, 0.784)
24 hours	0.805 (0.748, 0.857)	0.724 (0.673, 0.777)	0.719 (0.671, 0.779)	0.737 (0.670, 0.779)
MLP･LSTM				
6 hours	0.739 (0.678, 0.798)	0.656 (0.602, 0.717)	0.658 (0.605, 0.716)	0.655 (0.596, 0.726)
12 hours	0.736 (0.676, 0.797)	0.652 (0.600, 0.714)	0.655 (0.601, 0.715)	0.650 (0.593, 0.722)
24 hours	0.732 (0.671, 0.793)	0.646 (0.595, 0.710)	0.650 (0.596, 0.709)	0.643 (0.591, 0.717)
PT-LSTM-eICU				
6 hours	0.804 (0.747, 0.857)	0.708 (0.668, 0.771)	0.780 (0.729, 0.822)	0.666 (0.616, 0.720)
12 hours	0.801 (0.744, 0.855)	0.717 (0.666, 0.769)	0.716 (0.666, 0.77)	0.717 (0.665, 0.769)
24 hours	0.796 (0.738, 0.851)	0.713 (0.662, 0.767)	0.712 (0.661, 0.767)	0.714 (0.661, 0.766)
PT-MLP-eICU				
6 hours	0.793 (0.733, 0.847)	0.706 (0.656, 0.761)	0.712 (0.655, 0.763)	0.702 (0.656, 0.762)
12 hours	0.789 (0.729, 0.844)	0.703 (0.653, 0.759)	0.717 (0.652, 0.761)	0.695 (0.651, 0.761)
24 hours	0.782 (0.721, 0.839)	0.701 (0.647, 0.756)	0.708 (0.646, 0.756)	0.696 (0.646, 0.757)
PT-MLP･LSTM				
6 hours	0.727 (0.665, 0.789)	0.639 (0.573, 0.697)	0.605 (0.567, 0.703)	0.659 (0.560, 0.703)
12 hours	0.724 (0.661, 0.785)	0.622 (0.571, 0.693)	0.655 (0.596, 0.777)	0.602 (0.456, 0.670)
24 hours	0.718 (0.654, 0.779)	0.628 (0.554, 0.676)	0.625 (0.566, 0.698)	0.630 (0.560, 0.703)
∗(95% CI)				

Supplementary Table S4. Architecture and hyperparameters

Component	Pre-train model (30-day mortality)	MODS fine-tuning model (6/12/24h)
High-frequency inputs	7 vitals time-series (T=4 time steps, 4-hour window)	Same
Low-frequency inputs	34 low-frequency features (last time step used)	Same
High-frequency	Linear 7→32 + LayerNorm + Sigmoid +	Reuse pre-trained weights

Component	Pre-train model (30-day mortality)	MODS fine-tuning model (6/12/24h)
projection	Dropout(0.3)	
Sequence encoder	LSTM, hidden size 32, layers 8, dropout(0.3), batch_first=True	Reuse pre-trained weights
Post-RNN	LayerNorm + Linear 32→32 (take last time step)	Reuse pre-trained weights
Low-frequency MLP	10-layer MLP blocks: first 34→64, then 64→64 ×9; each block: Linear + BatchNorm + ReLU + Dropout(0.3)	Reuse pre-trained weights
Fusion	Concat (32 + 64 = 96)	Same
Shared FC trunk	8-layer FC blocks: 96→64, then 64→64 ×7; each block Dropout(0.3)	Reuse pre-trained weights (pre_fc)
Task head	Linear 64→2	Extra head: FC blocks 64→32, then 32→32 ×3, final Linear 32→3
Outputs	2 logits (binary mortality head)	3 logits (multi-label: MODS within 6/12/24h)

Supplementary Text S1: Model parameter settings

PT-MLP·LSTM-eICU

The model comprises two parallel branches — a temporal branch and a static branch. The temporal branch inputs are encoded using an 8-layer LSTM to capture latent temporal dynamics.The static branch, meanwhile, handles 34 laboratory and demographic variables, extracting high-level representations via a 10-layer fully connected network.

The outputs from both branches are fused in a 64-dimensional latent space, which is subsequently fed into a MLP to estimate the risk probabilities for the next 6, 12, and 24 hours.

During pretraining, the model is trained on a combined eICU–MIMIC dataset for a binary classification task (survival vs. death). The encoder parameters are then frozen, and fine-tuning is performed on the downstream prediction task.

Optimization is conducted using the AdamW optimizer (learning rate = 1×10^{-5}), with a batch size of 4096, for 300 epochs. The loss function is BCEWithLogitsLoss.

MLP·LSTM

This model consists of an 8-layer LSTM encoder (hidden size = 8) for time-series inputs, a 10-layer MLP (64 hidden units) for static data, and an 8-layer fusion MLP (64 hidden units) for multi-task output.

Training is conducted with AdamW (learning rate = 1×10^{-5}) for 300 epochs, employing early stopping to prevent overfitting.

PT-MLP-eICU

A pretrained dual-branch multilayer perceptron (PT-MLP) model was constructed, integrating 7 non-invasive and 34 invasive/static features. The pretrained encoders (8-layer and 10-layer MLPs) were fine-tuned using a three-output head to predict sepsis onset within 6, 12, and 24 hours.

The model was trained using AdamW (learning rate = 1×10^{-5}, weight decay = 1×10^{-3}) for 300 epochs, with early stopping enabled.

PT-LSTM-eICU

The PT-LSTM model combines a 12-layer pretrained LSTM encoder (hidden size = 128, dropout = 0.5) with a fine-tuned fully connected head (hidden size = 32, output size = 3) for multi-horizon sepsis prediction (6h, 12h, 24h).

Training was performed with AdamW (learning rate = 1×10^{-5}, weight decay = 1×10^{-3}) for 40 epochs, using BCEWithLogitsLoss as the objective function.

PT-MLP·LSTM

This architecture integrates an 8-layer LSTM encoder (hidden size = 8, dropout = 0.5) with a 10-layer MLP for static features (hidden size = 64). The two branches are fused through an 8-layer MLP (hidden size = 64, dropout = 0.3) to produce three outputs corresponding to 6h, 12h, and 24h prediction horizons.

The model was trained from scratch using AdamW (learning rate = 1×10^{-5}, weight decay = 1×10^{-3}) for up to 300 epochs, with BCEWithLogitsLoss as the loss function.

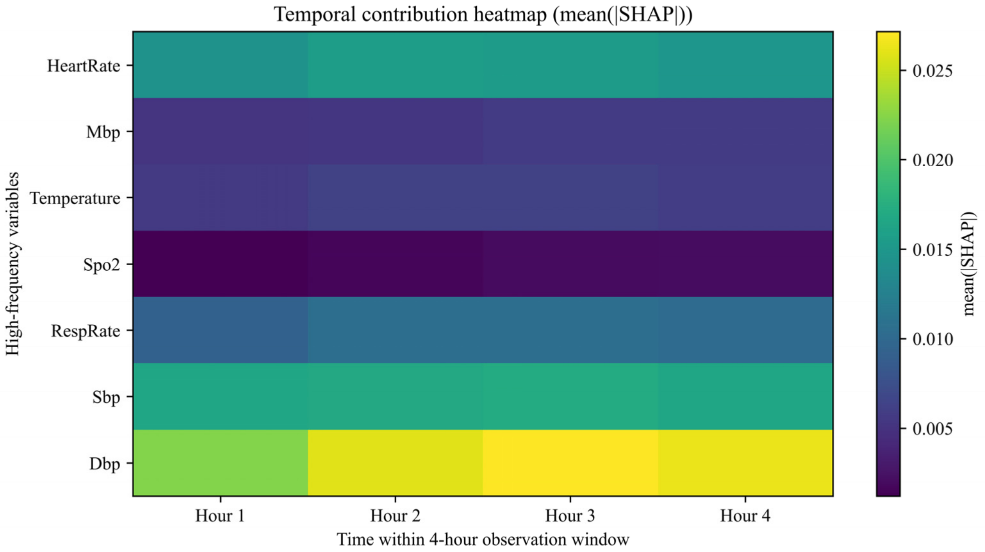

Supplementary Figure S1. Temporal contribution heatmap. The x-axis denotes four consecutive 1-hour segments (Hour 1–Hour 4), where Hour 4 is the segment closest to the prediction time. The y-axis lists high-frequency physiological variables. Colors represent the mean absolute SHAP value mean(|SHAP|) (averaged over the test set), with larger values indicating stronger contribution (higher importance) of a variable at a given hour to the model output.
